# Trans-Amplifying RNA: A Journey from Alphavirus Research to Future Vaccines

**DOI:** 10.3390/v16040503

**Published:** 2024-03-25

**Authors:** Ayşegül Yıldız, Cristian Răileanu, Tim Beissert

**Affiliations:** TRON—Translational Oncology at the University Medical Center of the Johannes Gutenberg University Mainz, 55131 Mainz, Germany; ayseguel.yildiz2@tron-mainz.de (A.Y.); cristian.raileanu@tron-mainz.de (C.R.)

**Keywords:** alphaviruses, defective interfering RNA, trans-replicon, self-amplifying RNA, trans-amplifying RNA, vaccine

## Abstract

Replicating RNA, including self-amplifying RNA (saRNA) and trans-amplifying RNA (taRNA), holds great potential for advancing the next generation of RNA-based vaccines. Unlike *in vitro* transcribed mRNA found in most current RNA vaccines, saRNA or taRNA can be massively replicated within cells in the presence of RNA-amplifying enzymes known as replicases. We recently demonstrated that this property could enhance immune responses with minimal injected RNA amounts. In saRNA-based vaccines, replicase and antigens are encoded on the same mRNA molecule, resulting in very long RNA sequences, which poses significant challenges in production, delivery, and stability. In taRNA-based vaccines, these challenges can be overcome by splitting the replication system into two parts: one that encodes replicase and the other that encodes a short antigen-encoding RNA called transreplicon. Here, we review the identification and use of transreplicon RNA in alphavirus research, with a focus on the development of novel taRNA technology as a state-of-the art vaccine platform. Additionally, we discuss remaining challenges essential to the clinical application and highlight the potential benefits related to the unique properties of this future vaccine platform.

## 1. Introduction

The recent success of SARS-CoV-2 mRNA vaccines exposed the profound potential of RNA therapeutics and created a broad awareness for the technology. However, challenges persist including side effects associated with innate immunity stimulation of the relatively high administration doses of 30 to 100 µg [1,2,3], and the need for repeated application to achieve durable immune responses. Efforts to reduce mRNA doses undertaken by vaccine developers often involve the usage of so called self-amplifying RNA (saRNA), an RNA format promising to minimize dosing mediated by autonomous intracellular multiplication of saRNA after transfer. Indeed, a newly licensed first saRNA-based vaccine appears to be highly effective upon injection of only 5 µg [4,5]. Additionally, it has been shown that intracellular saRNA amplification results in prolonged mRNA expression in animal studies [6], supporting the hope that saRNA may also reduce the need for repeated dosing.

**Figure 1:**
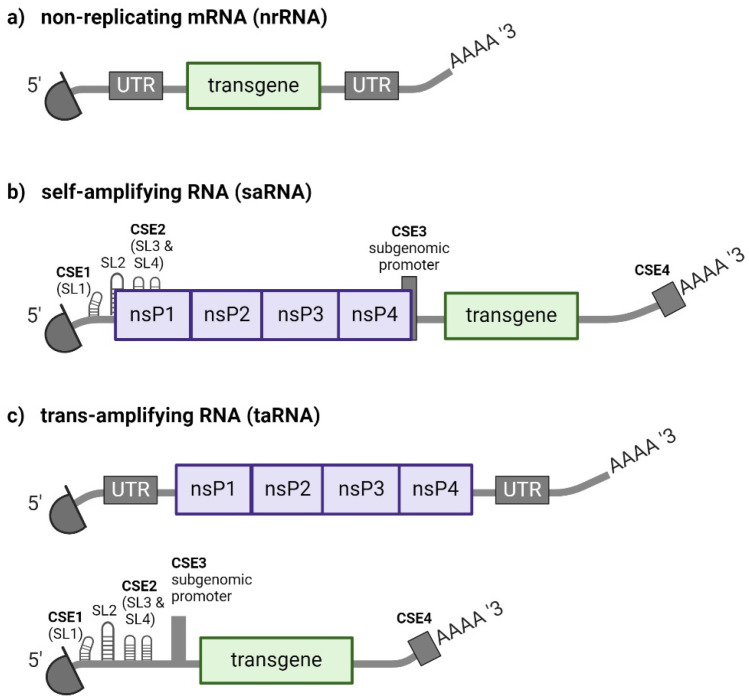
RNA vector platforms. (**a**) Non-replicating mRNA (nrRNA) consists of a transgene sequence flanked by 5′ and 3′ untranslated regions (UTR), a 5′ cap and a poly(A) tail. (**b**) Self-amplifying RNA (saRNA) contains the replicase gene composed of four non-structural proteins (nsPs) in the upstream position and a transgene downstream of the subgenomic promoter. It also has a 5′ cap and a 3′ poly(A) tail. saRNA possesses major conserved sequence elements (CSE), including stem loops (SL) that act as replicase recognition signals (adapted from [11]). (**c**) Trans-amplifying RNA (taRNA) comprises two separate RNA molecules: an nrRNA encoding the replicase and a transreplicon that encodes the transgene and contains the CSEs, ensuring its amplification by the replicase.

Upon cellular uptake, both mRNA and saRNA-based therapeutics remain exclusively within the cytoplasm where they are translated into protein. saRNA bares the important additional feature of transient replication. Essentially, therapeutic RNA does not enter the nucleus, is not reverse transcribed and therefore avoids potential insertional mutagenesis [7]. In terms of structure, mRNA and saRNA share common features such as a 5′ cap, as well as 5′ and 3′ untranslated regions (UTRs) and a 3′ poly-A tail. Furthermore, both encode an open reading frame (ORF) of a therapeutic transgene. The therapeutic gene is transiently expressed and limited by the natural degradation of the *in vitro* transcribed (IVT) RNA [8]. saRNA features a second ORF encoding the enzyme complex needed for RNA replication called replicase. In addition, saRNA incorporates a short internal subgenomic promoter (SGP) that essentially enables generation of a transcript of the therapeutic transgene that is translated separately (Figure 1a,b) [9]. Advancing this technology, our group recently introduced trans-amplifying RNA (taRNA) to the vaccine field. taRNA is a derivative of saRNA that is composed of two RNAs: a non-replicating mRNA (nrRNA) encoding the replicase and a so called transreplicon (TR) encoding the therapeutic transgene that is multiplied by the replicase [10]. In taRNA, the replicase nrRNA contains the structural features of mRNA, and the TR the structural features of saRNA except for a functional replicase ORF (Figure 1c). For taRNA to function effectively, the nrRNA that encodes replicase currently needs to be supplied in similar amounts of what is administered in mRNA vaccines. Nonetheless, a distinguishing feature of taRNA is that exceptionally low amounts of transgene coding TR are sufficient to elicit effective immune responses.

In this paper, we draw upon the history of transreplicating RNA discovery in alphavirus-infected cells and how understanding this RNA species continues to advance our knowledge on alphavirus biology. We review pioneering work unveiling so-called defective interfering particles (DIPs) and their exploitation, enabling generation of recombinant alphaviral particles. We highlight studies elucidating replicase functions and mechanisms of alphaviral replication before summarizing our work about how taRNA could be used as a vaccine. Finally, we provide an outlook on further work needed for optimization of taRNA and its clinical translation.

### Biology of Alphaviral RNA Replication

The basis of saRNA vectors originates from alphaviral genomes, which are a positive-sensed single stranded RNA encoding the viral structural and non-structural proteins (sPs and nsPs, respectively) as two coherent open reading frames separated by a SGP. Alphaviral replication is comprehensively reviewed elsewhere [12]. Upon their translation, the sPs encoded downstream of the SGP assemble to the viral shell. Engineering of saRNA vectors involves replacing sPs in alphavirus genomes which normally play essential roles in infectivity with transgenes of interest. The sequences encoding the four nsPs that together form the replicase polyprotein are retained to secure saRNA replication (Figure 1b). As a result, engineered saRNA replicates in cells by exploiting the alphaviral RNA replication machinery, enabling production of a high number of saRNA copies, which in turn facilitates enhanced gene expression without the release of infectious viral particles. Each of the four viral replicase subunits is essential to the saRNA system, executing specific functions during viral RNA synthesis [12,13]. Briefly, nsP1 functions as a membrane-anchored protein that localizes the replicase polyprotein to cellular membranes, leading to replication within membrane invaginations called spherules. Furthermore, nsP1 functions as an alphaviral capping-enzyme that adds a 5′cap to novel plus-strand RNA copies made in infected cells. nsP2 exhibits RNA triphosphatase, NTPase, helicase, and protease functions, supporting RNA replication and processing of the polyprotein. The roles of nsP3 are not fully understood, but it is well established that nsP3 associates with host proteins which either promote or inhibit RNA replication [14,15]. nsP4 is the RNA-dependent RNA polymerase of the replicase, constituting the core activity that eventually enables RNA amplification within cells. nsP4 also synthesizes the poly-A tail of novel plus-stranded RNA copies by its terminal adenylyl transferase activity.

The genomic RNA of alphaviruses contains sequence elements at the 5′ and 3′ termini that are highly conserved across alphavirus species. These so-called conserved sequence elements (CSEs) act as promoters for replicase to dock selectively onto the alphaviral saRNA and initiate genomic and subgenomic RNA-dependent RNA transcription. The CSEs were identified in the early 1980′s based on genome sequence comparison between alphaviruses and early RNA folding predictions. More precisely, a first CSE was found within the 5′-UTR of the alphaviral genome, and a second within the 5′-end of the nsP1 ORF named 52-nt CSE according to its length [16]. A third CSE located at the start site of the subgenomic RNA was identified as SGP [17,18]. Finally, a fourth CSE was identified as the 3′-terminal 19 nts [19] (Figure 1b).

**Figure 2:**
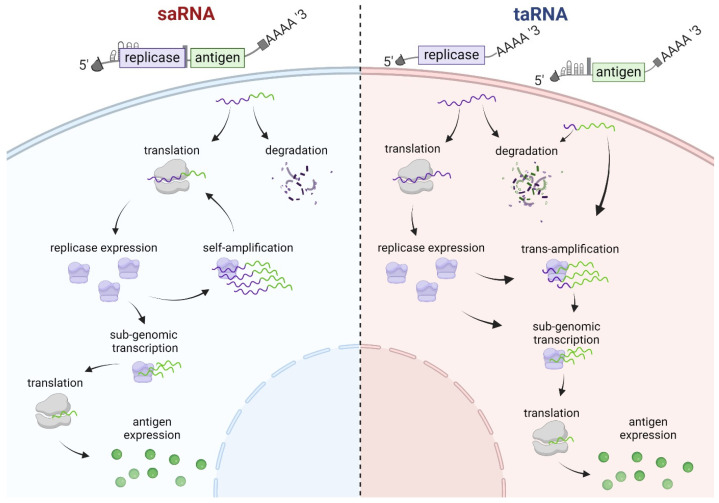
Schematic representation of self- and trans-amplifying RNA and replication mechanism. Once bicistronic saRNA is delivered into the cell (**left**), only the replicase is immediately translated. Replicase proteins direct self-amplification to generate genomic and subgenomic RNAs. Subsequent translation of replicase proteins can occur from the resulting genomic RNA while high antigen levels derive exclusively from the subgenomic RNA. Upon transfection with taRNA (**right**), replicase nrRNA is translated into replicase protein while concomitantly undergoing degradation alongside the TR RNA. Translated replicase proteins amplify genomic equivalents of the TR, as well as subgenomic transcripts from which translation of antigens occurs.

Importantly, cooperation of all four nsPs of the replicase with matching CSEs on the RNA template is required for saRNA amplification (Figure 2, left). Similarly, the replicase nsPs and sequence elements are required for amplification of the TR of taRNA. The decisive feature of taRNA is that these elements are separated into two RNAs. In taRNA, the replicase protein is generated by translation of replicase-coding nrRNA that itself is not amplified by replication because it lacks CSEs. However, replicase binding to CSEs deposited on the TR results in its amplification in *trans* (Figure 2, right) [10].

## 2. Natural Trans-Replicating RNA

Interestingly, trans-replicating RNA species can arise spontaneously in infected cells as a result of viral genome instability. RNA viruses are particularly prone to high mutation rates because viral RNA replicases do not contain proof-reading activity [20]. However, for viruses this genomic instability is not an evolutionary burden. It rather allows viruses to be remarkably adaptable helping them to persist and replicate within the complex physiological and immunological habitat of their host organisms, and to respond to changes in their environment. Besides point mutations, larger rearrangements and deletions of the genome can occur, giving rise to more or less largely truncated defective viral genomes (DVGs). These DVGs are unable to complete a replication cycle independently and therefore require the presence of a complementary helper virus with a full-length genome to compensate for compromised functions. Upon replication in the presence of a helper virus, DVGs can be packaged into viral particles that lack infectivity and are unable to establish a viral replication cycle. Because DVGs can potentially interfere with the formation of fully functional particles throughout the course of virus replication they have been coined “defective interfering particles” (DIPs) [21].

### 2.1. Investigation of Genome Sequence Requirements for Replication Using Defective Interfering Particles

In 1970, Huang and Baltimore proposed that the frequency of DIPs formation is significantly higher than what had previously been believed leading them to hypothesize that they might influence the course of viral diseases [21]. Subsequently, further studies began to provide evidence for the accumulation of DIPs through serial passaging of alphaviruses. These investigations not only shed light on the sequence requirements of the RNA genome essential for efficient replication by the replicase, but also offered insights into the molecular mechanisms underlying DIPs formation.

DIP accumulation was then observed during replication of Sindbis virus (SINV) in BHK-21 cells. Starting from a single plaque from the virus grown in chick embryo fibroblasts, the serial dilution of the virus on BHK-21 cells suggested the presence of DIPs by a significant drop of the hemagglutination titre and plaque-forming units (PFU) after several passages [22]. The idea was raised that DIPs could interfere with alphavirus replication during RNA synthesis simply because the DI RNA reduced in size would be more efficiently replicated compared to longer, unaltered virus RNA. In support of this idea, subsequent studies in various cell types, wherein DIP generation and propagation of Semliki Forest virus (SFV) was investigated, revealed that the formation of DIPs occurs at early passages. It was shown that the generation and replication of short DI RNA occurred at the expense of genomic RNA of SFV, as DI RNAs competed for the limited supply of virus RNA polymerase [23]. In most cell types, SFV DIPs evolved over time by a stepwise sequential deletion of internal regions of the standard virus genome while keeping 5′ and 3′ termini. These findings were important because they also suggested that the 5′ and 3′ terminal sequences of the alphavirus genome play a critical role for DI RNA and viral RNA replication. Subsequently, it was described that SFV DI RNA contains 106 conserved noncoding nucleotides important for replication or encapsidation located at its 3′ end that precede the poly(A) tail [24]. Sequencing of the 3′ end of genomic and subgenomic RNA of SINV, SFV and Middelburg virus confirmed that they all contained a highly conserved region of 20 nucleotides adjacent to the poly(A) tail [19]. More detailed insights into 5′ and 3′ end requirements were gathered by using a series of deletions spanning the entire SINV DI genome. For example, transfection of cells with these DI RNAs in the presence of helper SINV indicated that only sequences in the 5′-terminal 162 nucleotides and the 19 3′-terminal nucleotides are required for replication and packaging of these genomes [25]. The presence of highly conserved regions within the 5′ and 3′ termini suggested their potential recognition also by heterologous alphaviruses. Indeed, later it was shown that DIPs derived from wild-type SINV interfere with the replication of SFV and vice versa [26].

### 2.2. Engineered Defective Interfering RNAs to Introduce Foreign Genes into Cells

The alphavirus genome can serve as a stand-alone vector platform for gene expression. Along this line, the SINV RNA genome was successfully engineered by Xiong and colleagues to express the chloramphenicol acetyltransferase (CAT) gene downstream of the SINV subgenomic promoter. The resulting vector was shown to be self-replicating and enabled the expression of bacterial CAT in cultured insect, avian and mammalian cells. By supplying SINV structural genes *in trans*, vector genomic RNAs could be packaged into infectious particles that facilitate CAT expression, reflected by the production of recombinant alphaviral particles [27].

Later, it was demonstrated that SINV and its DIPs can be used as a bipartite vector to introduce foreign genes into cells [28]. The authors of this study replaced 75% of an SINV DI genome with foreign sequences, retaining a crucial 51-nucleotide segment at the 5′ end that is highly conserved in alphaviruses. Corresponding DI RNAs replicated in infected cells and were predominantly present at early passages. Notably, when the CAT gene was inserted into a DI RNA lacking this conserved region, DI RNAs’ amplification could not be detected, indicating the functional relevance of the 51-nucleotide sequence for DI RNA amplification. The findings suggested that DI RNAs could serve as vectors for introducing heterologous genes into cells.

## 3. Recombinant Alphavirus Production by Exploiting Defective Interfering RNA

### 3.1. Helper RNA

The efficient amplification and packaging of alphavirus DI genomes by a helper virus, coupled with their capacity to express foreign proteins, has paved the way for exploring and refining alphaviral vector systems. In the early 1990s, Peter Liljeström and Henrik Garoff pioneered this field by engineering SFV replicons to create a novel expression vector for mammalian cells [29]. They developed an *in vivo* packaging system for introducing recombinant RNA into cells through single round infectious viral particles (SRIPs). The recombinant RNA packaged into SRIPs contained transgenes downstream of the SGP, replacing the viral structural proteins while retaining the non-structural region of the SFV genome and the conserved 5′ and 3′ regions required for replication 3 *cis*. This recombinant RNA design equals the saRNA that is widely used today. For SRIP formation, they constructed defective ‘helper RNA’ based on DI genomes expressing the structural proteins, complementing the saRNA *in trans* to enable particle release. The helper RNA preserves essential 5′ and 3′ CSEs for helper replication and the entire structural region including the SGP. The SFV packaging signal located within nsP1 was conserved on the saRNA but deleted from the helper. Thereby, co-transfection of saRNA and helper RNA resulted in efficient SRIP production containing only saRNA but not helper RNA. Harvested and purified SRIPs efficiently transferred the saRNA to target cells leading to high transgene expression, while transfer and expression of helper-encoded structural proteins was avoided. The SFV system thus appeared to be a useful and safe method enabling the rapid production of recombinant virus stocks without recombination events leading to wild type SFV formation. Shortly thereafter, a comparable vector system was engineered for SINV [30]. Again, co-transfection of saRNA mediated replication and expression of structural proteins encoded by the defective helper RNAs. However, an encapsidation of defective helper RNAs was also observed despite the absence of a packaging signal. Overall, SINV saRNA transfer by SRIPs achieved comparable levels of transgene expression to the analogous SFV vector developed by Liljeström and Garoff [29].

### 3.2. Development of Advanced Helper RNA Systems

A few years later, Pushko et al. followed a similar approach to create an saRNA/helper RNA system from attenuated Venezuelan Equine Encephalitis virus (VEEV) [31], as with SFV and SINV expression vectors. However, their study noted the re-formation of live plaque-forming virus due to recombination events between the saRNA and the helper RNA. Since shortening of homologous sequence regions between both RNAs could not completely prevent recombination, the VEEV SRIPs system raised safety concerns. This problem could be solved by creating a bipartite helper system with one helper RNA molecule encoding the capsid and the other RNA molecule encoding the envelope proteins. Thereby, the required number of recombination events to reconstitute the genome of an infectious virus was increased, and upon co-transfer of VEEV saRNA with the two helper RNAs, the infectious virus was no longer detectable *in vitro* or *in vivo*. Additionally, immunization of mice with VEEV replicon particles elicited antibody responses against the expressed proteins. These developments essentially made the VEEV replicon system with the bipartite helper a compelling vaccine vector. A parallel study using SINV saRNA demonstrated that the co-transfection with two defective-helper RNAs facilitated high-titre SRIP production [32]. This system exhibited a high degree of safety, with no recombination events detected even after several passages. Later, Smerdou and Liljeström developed a comparable two-helper system based on the SFV RNA genome [33]. Similar to SINV, the SFV capsid integrated a translational enhancer at the 5′ end. The second helper RNA was constructed using the minimal enhancing sequence of the capsid followed by the 2A autoprotease from foot and mouth disease virus and the glycoprotein genes to express high levels of viral spike proteins. To further reduce the risk and probability of the formation of replication-competent particles, the helper RNA was engineered to carry a capsid gene with a mutation that abolishes self-cleavage capacity. This split system resulted in significantly improved safety and efficiency of packaging recombinant RNA expressing heterologous proteins.

The SRIP vector systems, comprising saRNA and helper RNAs, broadened the range of recombinant alphavirus vector applications, as potential vaccine vectors in particular (reviewed by [34]). Even though the safety of the SRIP platform increased considerably with the development of 2-helper RNA systems, concerns remained that the generation of replication-competent particles upon recombination was still occasionally detected. It was then found that SGP is required for either transgene or structural gene expression in both helper RNAs as well as the saRNA, which occasionally acted as a recombination site. A breakthrough to eliminate the concerns was thus achieved by generating helper RNAs devoid of SGPs [35]. These promoterless helper RNAs lack an SGP but still contain the viral 5′ and 3′ CSEs. Therefore, because the replicase generates only one type of transcript that is equivalent to the genomic RNA, a direct cap-dependent translation of the structural proteins is still possible. Remarkably, SRIP titres matched those produced with the previous helper RNA design. Moreover, promoterless helpers enhanced safety by introducing additional constraints on functional recombinants. In cell culture experiments, recombinants were not observed, affirming its safety. Finally, immunization of mice or non-human primates with VEEV SRIPs generated using promoterless helper RNAs encoding four vaccinia genes resulted in 100% survival upon challenge with the vaccinia virus and monkeypox virus. Essentially, this outcome solidified the SRIP vaccine platform as safe and effective.

## 4. Trans-Replicating RNA for Studying Alphaviral RNA Replication and Replicase Biology

The replication of alphaviral RNA relies on both expression of a self-maturating replicase and *cis*-acting CSEs of the RNA that adopt secondary structures interacting with replicase. Investigation of alphaviral RNA replication using full length viral genomes or saRNA is therefore cumbersome for two major reasons. First, the replicase coding region itself is amplified by the encoded replicase during replication which affects replicase expression levels. Replicase protein levels depend not only on the ongoing translation of transfected RNA copies but also on increasing RNA copy numbers. Thus, mutational studies aimed at altering replicase activity unavoidably result in alteration of replicase expression levels, which can complicate the interpretation of the results. Heterologous expression systems enabling the control of constant replicase expression levels, combined with RNA templates replicating *in trans*, help to study certain aspects of replicase biology more precisely. Second, alphaviral evolution resulted in condensed genomes wherein the *cis*-acting sequence elements overlap with the coding region of the replicase. More specifically, the 5′ end of the nsP1 gene overlaps with the promoter for genomic RNA replication, and the 3′ end of the nsP4 gene overlaps with the SGP (Figure 1). Consequently, mutational studies within these regions aiming to alter RNA structure often affect the amino acid sequence of replicase, inevitably leading to additional layers of complexity. The use of rationally designed trans-replicating DI-RNA (also referred to as transreplicons, TRs), wherein replicase is co-expressed by a second RNA, liberates these sequence elements from the constraint of maintaining a functional replicase ORF.

### 4.1. Defining RNA Promoter Requirements

Early approaches to identify the RNA elements required for replication relied mainly on sequence comparisons of alphavirus species, folding prediction and the assumption that sequence conservation was related to function [16]. Since CSEs were also found in DI RNAs, their significance for RNA amplification became even more obvious [36] and rational changes introduced into DI RNA further established this notion [18].

How the systematic and rational design of TRs can provide detailed insights into sequence requirements within the 5′ end of alphaviral RNA for transreplication research was demonstrated in a seminal study of Frolov et al. [37]. The authors investigated the impact of single and combined SL deletions of four stem loop structures (SL) within the 5′ CSEs of SINV and SFV, based on RNA-folding predictions indicating a similar sequence. They also explored mosaic constructs combining SLs of both viruses. While only SL1 located entirely within the 5′ UTR of both viruses, SL2 contained the replicase start codon and SL3 and SL4 encompassed the 51-nt CSE located within nsP1. Except for SL1, these deletions and SL exchanges would have drastically altered the nsP1 coding sequence in a viral genome or corresponding full-length saRNA, most likely abrogating replicase activity. However, the authors found that the 51-nt CSE was not absolutely required for replication but that it strongly enhanced replication. Replacement of SL1 in SINV-derived transreplicons with SL1 of SFV abolished replication, showing that replication primarily relies on SL1. Furthermore, a taRNA sharing structural similarities with the SINV 5′ UTR could functionally replace the SL1, similar to a previous observation made in DI RNA [38]. In a subsequent study, non-functional SINV-TRs due to an SL1 of SFV regained their replicative potential in response to the SINV replicase by extensions of the 5′ end [39]. Notably, the SFV replicase was overall more tolerant than the SINV replicase towards 5′ sequence changes. This is perhaps best illustrated by the ability of the SFV replicase to efficiently replicate SFV- and SINV-TRs, in contrast to the SINV replicase that can only replicate the SINV-TR [37]. Later, expression of TRs and comparable usage *in trans* by replicases derived from several alphaviruses revealed that the sequence and thermodynamic stability of the 5′ end SLs affect replication [40,41] and that a 5′ terminal dinucleotide AU conserved throughout alphaviral genomes [11] is crucial for replication [40,42]. Regarding sequence requirements at the 3′ end, the last 19-nt of the viral 3′ UTR (CSE4) preceding a poly(A) tail of a minimum of 11 residues could be defined as the core promoter driving minus-strand RNA synthesis across alphaviruses [43,44].

More recently, TRs have been more comprehensively exploited to assess to what extent alphavirus replicases can amplify TRs originating from another alphavirus species (cross-utilization) in human and mosquito cells [45,46]. Replicases of alphaviruses outside the SFV complex, including SINV, VEEV, Barmah Forest virus (BFV), and Eilat virus (EILV), preferentially replicated template RNA of the same virus and poorly cross-utilized others. Notably, the VEEV replicase could not replicate an SINV-TR. The SINV replicase could replicate the VEEV-TR but was unable to transcribe VEEV subgenomic RNA, likely because the VEEV SGP could not be recognized. Similarly, the SINV replicase could not replicate the Chikungunya virus (CHIKV)-TR, while the CHIKV replicase replicated an SINV-TR, highlighting non-reciprocal cross-utilization of templates by heterologous replicases [45]. The incompatibility of certain replicase/TR pairs was better understood upon employing chimeric TRs and targeted mutations. For replicases of SINV, CHIKV and Ross River virus (RRV), the ability of viral replicases to cross-utilize TRs of other viruses was defined by the very 5′ end, notably the first SL structure. Introducing mutations here significantly impacted TR replication [45]. Together, these findings were important because they essentially corroborated the notion that replicases recognize primary sequence motifs and associated RNA secondary structures to identify matching 5′ CSEs and SGP within the template RNA.

### 4.2. Investigating Replicase Subunit Functions

The replicase-encoding RNA initially undergoes translation as a single polypeptide chain (nsP1234), followed by sequential cleavage by the nsP2 protease at the boundaries between individual nsPs. This gives rise to the single nsPs and cleavage intermediates. First, nsP4 is cleaved from the precursor, leaving nsP123 untouched; next, nsP1 is released, and eventually, the remaining nsP23 intermediate is separated into nsP2 and nsP3 [47,48,49]. As much as understanding the mechanisms of trans-replication helped to elucidate the regulation of replication at the RNA level, it similarly helped to better understand the specific functions of the different replicase protein subunits and the activities of cleavage intermediates. For example, to this end, recombinant Vaccinia virus (VACV) was used in a number of studies to express replicase, single nsPs, or partially cleaved nsPs. TR replication was either studied *in vitro* using extracts of replicase-expressing cells or by co-transferring TRs into the cells [50,51,52]. The advantage of the heterologous expression of the replicase is that its abundance does not depend on RNA replication. Thereby, TR replication precisely reflects the activity of replicase or mutants, such as replicases with altered nsP2 cleavage sites preventing complete maturation. The synthesis of TR minus-strand copies, for instance, required the presence of nsP123 and nsP4, or of nsP1, nsP23 and nsP4, while fully cleaved nsPs no longer possessed the ability to synthesize minus strands [50,51,52,53]. Later, it was shown that full-length nsP4 isolated from infected cells was able to replicate both plus-strand and minus-strand templates, confirming its role as the alphavirus RNA polymerase [54]. Accordingly, nsP123 alone was not capable of RNA synthesis, nor was an inactive mutant of nsP4 in which a critical motif (GDD) was mutated.

A further development of taRNA research is the design of bicistronic TRs. Thereby, genomic replication and subgenomic transcription can be measured using reporter gene expression as a surrogate of RNA abundance by Northern blotting. More specifically, bicistronic TRs express a first reporter under control of the 5′ cap that indicates genomic replication and a second reporter from the subgenomic transcript that indicates subgenomic promoter activity [45,55,56]. With replicase being expressed *in trans* and itself not being amplified, such bicistronic TRs reflect replicase genomic and subgenomic transcriptional activity independent of replicase expression levels.

The expression of replicase *in trans* furthermore enabled replicase to express as two separate parts, with the nsP123 separated from nsP4. This was an important step because these two parts correspond to the first maturation step of replicase that acts as the minus-strand-specific replicase. Further attempts were then made to divide replicase even further into three components consisting of separate constructs for nsP1, nsP23 and nsP4. This separation essentially allows one to alter the stoichiometry of the components by transfecting different amounts of each nsP or to study chimeric replicases, wherein nsP123 originates from one and nsP4 from a heterologous alphavirus. These chimeras have been combined with TRs originating from the same selection viruses [57]. Using this strategy, the minus-strand replicases of SINV, CHIKV, O’nyong-nyong virus (ONNV), BFV, RRV, SFV, Mayaro virus (MAYV), VEEV and EILV were found to exhibit comparable activities to full-length replicases produced as a single polyprotein. Chimeric replicases remained highly active when nsP123 originating from viruses of the SFV complex was combined with nsP4 originating from alphaviruses of different complexes. In contrast, the nsP123 components of VEEV and SINV formed functional replicases solely when nsP4 belonged to the homologous virus. Interestingly, applying these findings to full-length virus genomes, chimeras of SINV harbouring the nsP4 from CHIKV, ONNV, BFV, RRV, SFV, MAYV, VEEV, or EILV, were not viable at 37 °C but replicated at 28 °C. When combining P123 and nsP4 from different viruses within the SFV complex, pairings in which both nsP4 and the template RNA originated from the same virus exhibited elevated levels of subgenomic RNA synthesis. This observation emphasizes the significant role of the nsP4 component in recognizing and efficiently utilizing the SGP [57].

Further studies enabled by trans-replicating systems including experiments involved the insertion of epitope tags into nsPs or fusion with fluorescent reporter genes [56,58]. Additionally, targeted point mutations within active sites benefit from the heterologous expression of replicase and reporter expressing TRs [47,59,60,61]. Overall, trans-replicating systems are invaluable for systematically analyzing replicase modifications.

### 4.3. Investigating Spherule Formation

Viral RNA replication triggers innate immune mechanisms of cells and organisms aiming to inhibit virus propagation. In insect cells, dsRNA-mediated RNA interference (RNAi) is the major antiviral defence system [62], whereas mammalian cells activate their innate immune system leading to interferon release and subsequently the upregulation of a large number of antiviral genes [63]. Alphavirus replicases can evade innate immunity responses and facilitate efficient replication, by unloading the replication machinery to cellular membranes upon infection [64]. This results in the formation of micro-compartments called spherules, which are small bulb-shaped membrane invaginations housing viral replication complexes (RC) made of nsPs and RNA templates. Remarkably, the spherule lumen connects to the cytoplasm by a narrow channel, allowing the influx of nucleotides and the efflux of novel single stranded RNAs, while dsRNA replication intermediates remain within the spherule lumen [65,66,67]. Recently, the structural organization of alphaviral spherules was disclosed by high-resolution cryo-electron microscopy and tomography [68,69].

During the process of elucidating spherule formation, trans-replicating systems enabled researchers to connect replicase processing and RNA replication especially to spherule formation. It was first suggested that unprocessed nsP123 and dsRNA replication intermediates are necessary for spherule formation [70]. Various SFV replicase protein and TR constructs then helped to dissect the distinct stages in functional RC assembly [56], still suggesting that replicase proteins alone or a polymerase-deficient replicase fail to form spherules. More recently, spherule formation was observed independent of replicase activity or template RNA availability [71]. Upon blocking of nsP2 cleavage sites of the replicase, partially processed replicase alone generated spherule structures, and so did an inactive replicase mutant. Intriguingly, all four nsPs were shown to be essential for spherule formation, while replicase maturation should not be fully completed. Cleavage of nsP4 from the replicase precursor was necessary, while P123 (more efficient than nsP1 + nsP23) needed to remain uncleaved [71]. Thus, replicase intermediates capable of synthesizing negative-strand RNA [72] are required to initiate spherules. In the absence of template RNA, spherules exhibited a tendency to be smaller. The size of spherules is correlated with template RNA length, as transreplicons of two different sizes produced two distinctly sized spherules [55]. This suggests spherule size by itself does not affect replicase function and that one template molecule is incorporated into an individual spherule.

Overall, significant advances have been made over decades of alphaviral research with the help of trans-replicating RNA, and the use of trans-replicating RNA will certainly continue to advance our understanding of alphaviral biology.

## 5. Trans-Amplifying RNA Vaccines

In several of the aforementioned studies, reporter genes were encoded by the transreplicons. Even though the expression levels of the transreplicons were not the primary focus of these studies, their expression levels were often very high [55,56,73]. Furthermore, the production of recombinant viral particles using helper RNA-encoding alphaviral structural genes was efficient, which indicated robust expression levels of the sPs from the helper RNA. Essentially, these observations, when combined, raised the idea that TRs could bear the potential to be used as an RNA-based vaccine.

A prerequisite for an RNA-based vaccine is that all required proteins are encoded on IVT RNA and delivered simultaneously. This approach was undertaken in a study that revisited the essential sequence elements required for alphaviral replication using a large set of TRs (named splitzicons) [73]. This study discussed a potential utilization of TRs as a vaccine. In a proof-of-concept study, we were then the first to explore antigen-coding TRs together with replicase-coding RNA provided *in trans* as a vaccine. We found that TR expression using replicase encoded on nrRNA was more efficient than replicase encoded on saRNA [10]. This all-RNA platform was named trans-amplifying RNA (taRNA), distinct from saRNA or the experimental use of trans-replicating RNA systems. When immunizing mice, a combination of 20 µg replicase nrRNA and 50 ng transreplicon-encoding influenza HA mounted HA-specific immune responses comparable to 20 µg conventional mRNA-HA or 1.25 µg saRNA-HA. The required dose of antigen-coding TR was strikingly 400x less TR than replicase nrRNA, highlighting the advantage of this strategy. Overall, this meant that the amount of TR was practically negligible compared to the total RNA dose. Moreover, the finding that nrRNA is a more suitable option for delivering replicase compared to saRNA simultaneously implied that deleterious RNA recombination would be unlikely. Indeed, a follow up study using a CHIKV taRNA revealed that nrRNA replicase and TRs do not recombine [74]. In this study, which introduced taRNA as a vaccine candidate against CHIKV, the researchers demonstrated the high amplification of TR and antigen expression *in vitro*, without recombining to replication-competent CHIKV. Importantly, the taRNA-based vaccine induced humoral and cellular immune responses in a mouse model and provided protection against CHIKV challenge infection. Further observations made in this study confirmed previous studies that longer TR-RNA templates amplified slower and reached lower total RNA levels compared to shorter ones [55,56]. In the same year, the researchers investigated the feasibility of a bivalent vaccine candidate [75]. Here, two TR-RNAs encoding CHIKV and RRV envelope proteins were used, demonstrating efficient co-amplification and high antigen co-expression. Immunization of mice induced specific humoral and cellular immune responses against both CHIKV and RRV. However, antibody titres and the neutralization capacity were higher after immunization when using a single TR-RNA, suggesting potential immune interferences or dosing effects in the bivalent vaccine candidate. Interestingly, alphavirus-specific T cell responses remained equally potent after bivalent vaccination. This seminal study provided important insights into the design of multivalent taRNA-based vaccines, expanding their potential applications.

**Figure 3:**
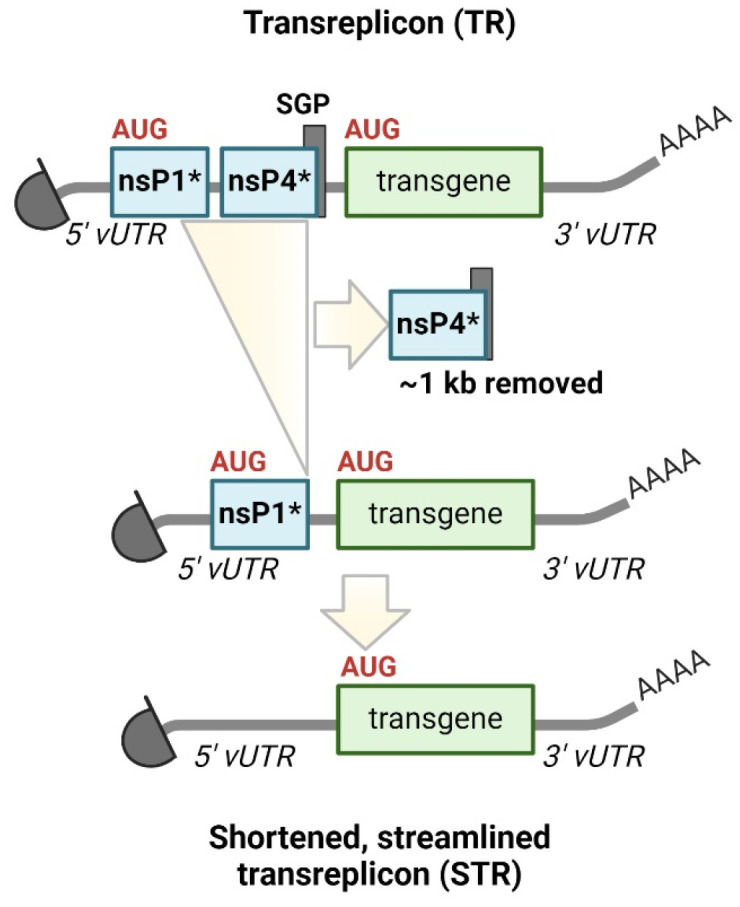
Structure and simplification of transreplicon. Shortened and streamlined transreplicon (STR) was created by deleting the original as well as all putative start codons within 5‘ CSE/nsP1 of the transreplicon (TR) and by removing the nsP4-subgenomic promoter (SGP). The AUG-codon mutations in the STR construct prevent a putative nsP1-peptide translation, thus, STR replication no longer supports subgenomic transcription. Instead, transgenes encoded on the STR are translated directly from original *in vitro* transcribed STR RNA if capped, and from positive-sensed STR copies resulting from intracellular replication. nsP1*: 5′ end of nsP1 containing the CSE; nsP4*: 3′ end of nsP4 including the SGP [76].

Because the first generation of taRNA proved efficient in the PoC study, we recently generated strategies to optimize the taRNA platform by simplifying the structure of the TR [76]. The TR simplification involved removing remnants of the replicase gene, the SGP and subgenomic transcript formation, resulting in a shortened TR (STR) closely resembling conventional mRNA. Notably, the STR retained alphaviral RNA promoters within the UTRs (Figure 3). STRs resemble promoterless helper-RNA [35], albeit with additional 5′ CSE modifications. By eliminating the original start codon and further AUG triplets, the start codon of the desired antigens became the most 5′-AUG, resulting in bypassing the translation of peptides corresponding to the replicase nsP1 N-terminus. Compensating mutations were introduced to preserve the structure and function of the 5′ CSE, while the SGP was eliminated without replacement. Transgenes encoded on the STR became accessible for translation from the 5′ cap. Unlike previous TRs, and similar to promoterless helper-RNAs, STRs no longer form subgenomic RNA species upon replication, producing only genomic replication products. To enhance STR amplification, a directed evolution strategy was employed, resulting in the accumulation of faster replicating mutant templates characterized by an extended 5′ end. Moreover, the flexibility of the taRNA platform was highlighted by the successful replication of various heterologous STRs by the SFV replicase, including Forth Morgan virus, Aura virus, Highlands J virus, Madariaga virus and CHIKV, thereby confirming findings in the literature [45,57].

Together these studies demonstrated the profound adaptability of the taRNA platform and its potential use for vaccinations against infectious diseases.

## 6. Future Perspective of taRNA Vaccines, Challenges and Open Questions

The hallmark of taRNA, the combination of two RNAs with different functions, opens up opportunities and poses particular challenges. The replicase-encoding nrRNA is the engine of the system and drives the expression of the antigen-encoding STR. As described above, replicase only requires CSEs at the ends of the STR, while it is indifferent to the sequences of the antigen ORF. The replicase is also indifferent to whether one, two or more antigens are encoded by different CSE-flanked STRs. Overall, the results of previous taRNA vaccine publications indicate that a much greater mass of replicase nrRNA than STR is required. Whether a taRNA vaccine will eventually require a lower total mass of both RNAs compared to the mass of modified non-replicating mRNA or saRNA still requires testing in larger animal models and humans. Probably further molecular optimization of the STR and the replicase is required to achieve this goal. Irrespective of the outcome of these studies, the bipartite nature of taRNA can be exploited to develop a replicating RNA vaccine with low immunogenicity, to assemble multivalent vaccines, or to prepare for future pandemics.

### 6.1. Towards a Non-Immunogenic taRNA Vaccine

Vaccines often cause immediate symptoms related to the innate immune response to vaccination, commonly referred to as reactogenicity, which ultimately limits the dose that can be administered. mRNA vaccines are more reactogenic when prepared from unmodified nucleotides, which was observed in clinical trials of SARS-CoV-2 vaccines [77,78]. The approved and successfully administered mRNA vaccines were modified with the nucleotide N1-methylpseudouridine (N1mΨ) instead of uridine. This greatly reduced the ability of the mRNA to activate innate immunity, subsequently reactogenicity, and the vaccines were well tolerated by the majority of vaccinated individuals [79,80]. Given this great effect of RNA modification, it was naturally considered for saRNA as well. Unfortunately, it was found that saRNA loses its function when modified by N1mΨ [81]. Accordingly, the recently approved saRNA vaccine does not contain modified nucleotides. However, its low dose of only 5 µg probably reduced reactogenicity to such an extent that it became tolerable [4,5]. Encouragingly, a recent comprehensive test of a variety of modified nucleotides has shown that 5-methyl cytidine (5 mC) reduces the immunogenicity of saRNA without affecting its function [82,83], raising hopes for even less reactogenic saRNA in the future.

The taRNA offers a unique advantage for the incorporation of modified nucleotides. The nrRNA encoding the replicase can accept all modifications that reduce immunogenicity without affecting RNA translation. Modified nucleotides, similar to saRNA, impede STR replication. Notably, N1mΨ exerts a potent inhibitory influence, while 5 mC allows replication (own unpublished observation). Since nrRNA replicase is used in excess, most of the RNA mass of taRNA may benefit from the clinically tested N1mΨ modification. The STR RNA could remain unmodified, with the total amount of unmodified RNA likely to remain below the threshold of unacceptable reactogenicity. Alternatively, the STR could be provided with 5 mC or another modification that may be developed for saRNA in the future. The production of a low-immunogenic taRNA is therefore already easily possible today, and the remaining immunogenicity could be further reduced in the near future.

### 6.2. Realizing Multivalent Vaccines Using taRNA

The CSEs located at the 5′ and 3′ ends of an RNA determine whether or not an RNA is a template for amplification by replicase, while the coding sequence inserted between these ends should not affect replication. Nevertheless, if in rare cases the coding sequences negatively affect replication, e.g., by intramolecular RNA interaction over larger distances, the use of synonymous codons should solve the problem. Overall, the indifference of the replicase to the coding sequence of the STR should allow the amplification of STR mixtures, which is the prerequisite for the construction of multivalent vaccines. The replication rates of STRs, however, could vary with the length or individual complexity of RNA folding of the different coding sequences. Therefore, further studies are needed to discover whether there are universal rules for determining the TR stoichiometry of a multivalent taRNA vaccine or whether each multivalent vaccine requires individual optimization of TR stoichiometry. Regardless of the ratio of the different TRs to each other, the total RNA amount of a multivalent taRNA vaccine will most likely largely depend on the amount of nrRNA replicase, similar to mono- and bivalent taRNA vaccines tested in mice [74,75]. This is in marked contrast to multivalent mRNA vaccines. Here, for each antigen encoded on a single mRNA molecule, the total amount is increased by the amount of mRNA required for a robust immune response against each of these antigens [84]. At an overall lower dose level, this is also likely to be the case for multivalent saRNA vaccines. Therefore, multivalent RNA vaccines run the risk of quickly exceeding the maximum tolerated dose, unless each antigen-encoding RNA only slightly increases the dose—as is the case with taRNA.

### 6.3. taRNA to Accelerate Seasonal Vaccine Production and Improve Pandemic Preparedness

As mentioned above, the two-part design of taRNA is the main difference from other nucleic acid vaccines. Replicase nrRNA as the main component of the vaccine not only enables low-immunogenic replicating RNA vaccines or facilitates multivalent vaccines, but can also accelerate the production of vaccines. Let us assume that a future vaccine will contain 9 times more replicase nrRNA than TR-RNA. This would mean that 90% of the RNA vaccine mass does not code for the antigen. The replicase nrRNA could therefore be produced independently of the antigen-coding STRs and stored until use. We believe that this will be invaluable in two particular situations. First, in the production of seasonally adapted tetravalent influenza vaccines. 90% of the vaccine—i.e., the mass that would correspond to the replicase nrRNA—would be available from stock before it is known which strains of influenza viruses need to be selected for the vaccine. The STRs that code for the hemagglutinin of these strains make up the remaining 10% of the vaccine, and only these would need to be produced in time for the start of the vaccination campaign. The cost of producing large batches of the flu vaccine each year would be much lower. Seasonally updated vaccines against SARS-CoV-2 would benefit in a similar way.

In addition, the replicase nrRNA in stock would also be immediately available should an emerging virus trigger a pandemic. The time needed to develop viral antigens and test them for their suitability as a vaccine could be used to produce even more replicase nrRNA. Apart from possible difficulties in finding suitable antigens for a vaccine, most of the required mRNA mass for a first large batch of a vaccine would already be available at the time of approval. The speed with which a completely new vaccine could be made available would benefit enormously from this approach.

### 6.4. taRNA Formulation

Therapeutic RNA must be formulated for effective delivery to humans. COVID-19 mRNA vaccines, for example, use LNPs for optimal delivery. The efficacy of taRNA will likely heavily depend on optimization of formulations, as both RNA species of taRNA must be delivered together to the same cell to be functional. A cell transfected only with replicase nrRNA cannot contribute to antigen-specific immune responses, and a cell receiving only STR will at best express basic levels of the antigen. Even with a monovalent taRNA vaccine, the question already arises whether it is preferable to mix the RNA before formulation (co-formulation) or to formulate both RNAs separately and mix the encapsulated RNAs shortly before administration. *In vitro* experiments with commercial liposomes indicate that both methods lead to transreplication, but in our experience, co-formulation with liposomes leads to higher transfection rates (own unpublished data). On the other hand, a separate formulation may offer more flexibility, especially for a vaccine that requires unanticipated adaptation due to the rapid emergence of a new strain. As long as the two RNAs are not mixed, the STR could be changed in the short term. Therefore, both strategies should be carefully evaluated and compared in terms of their potency and efficacy, as well as their feasibility in an industrial setting. Ultimately, the decision on which approach to pursue for a particular indication will involve both scientific and economic considerations.

## 7. Concluding Remarks

In light of the literature summarized in this manuscript, it is fascinating to observe the significant contributions made by naturally occurring defective interfering RNA and engineered transreplicons to the advancement of alphaviral research over the past 50 years. They have not only been pivotal in shaping our understanding but have also served as gene transfer tools for over three decades. With their recent adaptation as vectors for antigen delivery within taRNA, transreplicons have entered the field of nucleic acid-based vaccines, holding promise as potent tools in the fight against future infectious threats. While writing this review, the first saRNA vaccine was licenced in Japan [4], following the successful path of COVID-19 mRNA vaccines. Although taRNA is yet to undergo clinical translation, our optimism is grounded in sound reasons, anticipating its eventual success.
